# Renal Dysfunction is a Risk Factor of Death after Gastric Endoscopic Submucosal Dissection in Elderly Patients Aged ≥80 Years

**DOI:** 10.1155/2019/7145182

**Published:** 2019-09-09

**Authors:** Kenichiro Okimoto, Makoto Arai, Hideaki Ishigami, Takashi Taida, Keiko Saito, Daisuke Maruoka, Tomoaki Matsumura, Tomoo Nakagawa, Tatsuro Katsuno, Naoya Kato

**Affiliations:** Department of Gastroenterology, Graduate School of Medicine, Chiba University, Chiba, Japan

## Abstract

**Introduction:**

Endoscopic submucosal dissection (ESD) for early gastric cancer (EGC) is well accepted. However, its adaptation for elderly patients is unclear. This study aimed to investigate the prognosis and long-term outcomes of ESD for EGC in elderly patients aged ≥80 years by comparing their findings to the findings of patients aged <80 years.

**Materials and Methods:**

The study included 533 patients (632 lesions). The patients were divided into an elderly group (age, ≥80 years; 108 patients; 128 lesions; mean age, 83.4 ± 2.7 years) and a nonelderly group (age, <80 years; 425 patients; 504 lesions; mean age, 69.6 ± 7.9 years). We compared patient and lesion characteristics, overall survival (OS), and disease-specific survival (DSS) between the 2 groups retrospectively. Multivariate analysis was performed to clarify the risk factors of death after ESD.

**Results:**

The rate of curative resection and adverse events was not significantly different between the groups. The mean survival time periods with regard to OS/DSS in the elderly and nonelderly groups were 75.8 ± 5.9 and 122.8 ± 2.6 months (*P* < 0.05)/120.0 ± 3.0 and 136.4 ± 0.6 months (not significant), respectively. In the elderly group, eGFR <30 ml/min/1.73 m^2^ was an independent risk factor of death (hazard ratio = 5.32; 95% confidence interval = 1.39–20.5; *P*=0.015).

**Conclusion:**

ESD for EGC can be performed safely and can achieve high curability with good prognosis in elderly patients aged ≥80 years. After ESD, close attention should be paid to elderly patients with severe chronic kidney disease.

## 1. Introduction

Gastric cancer is one of the most common cancers globally and is the second most common cause of cancer-related death [1]. Among the various treatment options for early gastric cancer (EGC), gastric endoscopic submucosal dissection (ESD) is well accepted because it is less invasive than conventional surgery [2–5]. The guidelines of ESD for EGC have been established [6]. Good short- and long-term outcomes of gastric ESD have been reported when the procedure was conducted following the guidelines [7–13].

Recently, the life expectancies of male and female patients have been reported to be 79 and 86 years in Japan [14], indicating that the aging society is rapidly growing. Elderly patients tend to have several comorbidities, and their general condition is occasionally poor, indicating that minimally invasive treatments for EGC, such as ESD, in these patients are important. However, we are sometimes distressed by the adaptation of gastric ESD for elderly patients when their clinical conditions are considered. Although several reports have mentioned the outcomes of gastric ESD in elderly patients [15–20], there is little consensus on the indication of gastric ESD and the prognosis in elderly patients.

The aim of this study was to investigate the prognosis and long-term outcomes of gastric ESD in elderly patients aged ≥80 years by comparing their findings to the findings of patients aged <80 years.

## 2. Methods

### 2.1. Study Design and Patients

This retrospective study was conducted in Chiba University Hospital, Japan. From May 2005 to September 2016, 533 patients (632 lesions) who underwent gastric ESD for EGC were enrolled. We classified patients aged ≥80 years into an elderly group and those aged <80 years into a nonelderly group according to the previous report [21]. There were 108 patients (128 lesions) in the elderly group and 425 patients (504 lesions) in the nonelderly group. We compared patient and lesion characteristics, as well as long-term outcomes between the 2 groups. Curability was assessed, and intragroup comparisons were performed. The study protocol was approved by the Institutional Review Board of Chiba University and was registered (Clinical Registration Number: UMIN000024856).

### 2.2. Indication of Gastric ESD

We performed ESD for EGC that was judged as the absolute or expanded indication before ESD, according to the Japanese gastric cancer treatment guidelines 2010 (ver. 3) [6]. Patients with ECOG performance status (PS) 0 or 1 were treated. When the patients had cancers other than EGC, we consulted with doctors from specific fields, and only if their prognosis was considered acceptable on the condition that curative ESD was achieved, we performed ESD. When patients had other comorbidities, we held discussions among individuals from our ESD treatment team, as well as other specific fields, and physicians closely judged the adaptation of gastric ESD.

### 2.3. Patient and Lesion Characteristics

We collected patient clinical data, including age, sex, body mass index (BMI), preoperative estimated glomerular filtration rate (eGFR), and comorbidities (cardiovascular disease (CVD), history of cerebrovascular disease, and cancers other than gastric cancer). Adverse events of ESD were also assessed. The locations of the lesions in the stomach were classified into upper, middle, or lower. Tumor size, pathological findings, and depth of invasion were examined along with the Japanese gastric cancer treatment guidelines [6]. Macroscopic type was classified into elevated, flat/depressed, or mixed (combination of the previous 2 types), as previously reported [20]. We compared these factors between the 2 groups.

### 2.4. Curative Evaluation of the Resected Specimens

Curability was examined according to Japanese gastric cancer treatment guidelines [6]. Curative resection (CR) was determined when the resected specimen with negative horizontal and vertical margins and without lymphovascular invasion met any of the following 4 criteria: (1) differentiated-type intramucosal adenocarcinoma without ulcerative findings regardless of the tumor size; (2) differentiated-type intramucosal adenocarcinoma with ulcerative findings and tumor size ≤30 mm; (3) differentiated-type shallow submucosal (SM1) carcinoma and tumor size ≤30 mm; (4) undifferentiated adenocarcinoma without ulcerative findings and tumor size ≤20 mm.

Non-CR was determined when the resected specimen did not meet any of the 4 CR criteria or was margin (horizontal or vertical) positive.

### 2.5. Long-Term Outcomes

We collected data regarding long-term outcomes. For patients who visited our hospital regularly, we obtained data from the databases in our hospital. For patients who did not visit our hospital regularly, we contacted the patients at their home or obtained information from their physician. We assessed overall survival (OS) and disease-specific survival (DSS) with regard to gastric cancer for each group and compared OS and DSS between the elderly and nonelderly groups. Survival rates between patients whose eGFR was <30 ml/min/1.73 m^2^ (CKD stage G4 and G5 according to Japanese CKD guidelines [22]) and other patients in each group were analyzed. Also, survival rates between patients whose BMI was <22 kg/m^2^ (BMI 22 kg/m^2^ is Japanese ideal BMI [23]) and other patients in each group were analyzed. Additionally, univariate and multivariate analyses in each group as well as overall assessments between patients who died and those who were still alive during the observation period were performed.

### 2.6. Statistical Analysis

Patient and lesion characteristics, curability, adverse events, and items with regard to long-term outcomes were compared between the elderly and nonelderly groups using Fisher's exact test, the chi-square test, or unpaired *t*-tests. OS, DSS, and the survival rate were investigated using the Kaplan–Meier method and were compared using the log-rank test. OS and the survival rate were calculated from the first ESD to the date of death or latest confirmation of patient survival. DSS was calculated from the first ESD to the date of death caused by gastric cancer or latest confirmation of patient survival. For calculating DSS, patients who died from conditions other than gastric cancer were excluded. Univariate and multivariate analyses were performed with cox-regression analysis. Variables with *P* < 0.1 in univariate analysis were subjected to multivariate analysis. All statistical analyses were performed using SPSS version 22.0 (SPSS Inc., Chicago, IL, USA). A *P* value <0.05 was considered statistically significant.

## 3. Results

### 3.1. Patient and Lesion Characteristics

Patient characteristics are shown in Table 1. The mean ages of the patients in the elderly and nonelderly groups were 83.4 ± 2.7 and 69.6 ± 7.9 years, respectively. There were no significant differences with regard to sex, BMI, and ECOG-PS between the 2 groups. Preoperative eGFR (ml/min/1.73 m^2^) was significantly lower in the elderly group than in the nonelderly group (57.6 ± 16.8 vs. 69.4 ± 18.9, *P* < 0.05, unpaired *t*-test). The percentage of patients who had already started using anticoagulants and/or antiplatelet drugs was significantly higher in the elderly group than in the nonelderly group (27.8% vs. 16.0%, *P* < 0.05, chi-square test). With regard to comorbidities, the number of patients with hypertension was higher in the elderly group than in the nonelderly group (50.0% vs. 38.4%, *P* < 0.05, chi-square test).

Table 2 shows the characteristics of the lesions. There were no significant differences with regard to macroscopic type, location, histological type, tumor depth, tumor size, and lymphatic vessel invasion between the elderly and nonelderly groups. On the other hand, the ratio of venous invasion was significantly higher in the elderly group than in the nonelderly group (4.7% vs. 1.0%, *P* < 0.05, Fisher's exact test).

### 3.2. Curability and Adverse Events Related to ESD

The details of curability and adverse events are summarized in Table 3. There were no significant differences with regard to en block resection and CR between the elderly and nonelderly groups. Additional surgical resection was performed in 2 (1.9%) patients from the elderly group and 24 (5.6%) patients from the nonelderly group. In the nonelderly group, 3 out of 24 patients had residual tumor in their resected specimen and no patient had lymphatic metastasis. In elderly group, one patients had lymphatic metastasis as well as tumor recurrence in residual specimen (details of this patient are shown in Supplementary [Supplementary-material supplementary-material-1]). The rates of adverse events related to ESD, such as postoperative bleeding, perforation, aspiration pneumonia, and delirium, were not significantly different between the 2 groups. There were no deaths related to adverse events, and every adverse event was reversible.

### 3.3. Long-Term Outcomes of ESD

The long-term outcomes of ESD are shown in Table 4. The mean observation periods were 26.9 ± 23.2 and 36.4 ± 29.6 months in the elderly and nonelderly groups, respectively (*P* < 0.05, unpaired *t*-test.). The rate of the patients with ≥3 years follow-up was 28.7% and 41.9% (31 and 178 patients) in the elderly and nonelderly groups, respectively. The rate of the patients with ≥5 years follow-up period was 7.4% and 20.0% (8 and 85 patients) in the elderly and nonelderly groups, respectively.

**Figure 1:**
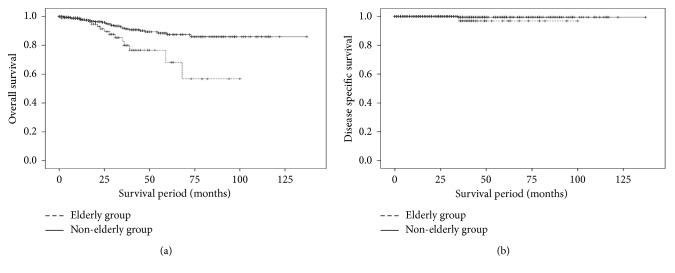
Comparison of overall survival (OS) and disease-specific survival (DSS) between the elderly and nonelderly groups. (a) Comparison of OS between the elderly and nonelderly groups. The mean survival time periods were 75.8 ± 5.9 and 122.8 ± 2.6 months in the elderly and nonelderly groups, respectively (*P* < 0.05, log-rank test). (b) Comparison of DSS between the elderly and nonelderly groups. The mean survival time periods were 98.0 ± 2.0 and 136.4 ± 0.6 months in the elderly and nonelderly groups, respectively (not significant, log-rank test).

Each group had 1 gastric cancer death. Within the observation period, 13 (12.0%) and 28 (6.6%) patients in the elderly and nonelderly groups, respectively, died from conditions other than gastric cancer. The mean time periods from ESD to death from conditions other than gastric cancer were 30.2 ± 16.6 and 26.8 ± 17.8 months in the elderly and nonelderly groups, respectively. The rate of patients who died from renal dysfunction was significantly higher in the elderly group than in the nonelderly group. OS in each group is shown in Figure 1(a). The mean survival time periods were 75.8 ± 5.9 and 122.8 ± 2.6 months in the elderly and nonelderly groups, respectively (*P* < 0.05, log-rank test). DSS is shown in Figure 1(b). The mean survival time periods were 98.0 ± 2.0 and 136.4 ± 0.6 months in the elderly and nonelderly groups, respectively (not significant, log-rank test).

### 3.4. Univariate and Multivariate Analyses of Risk Factors

**Figure 2:**
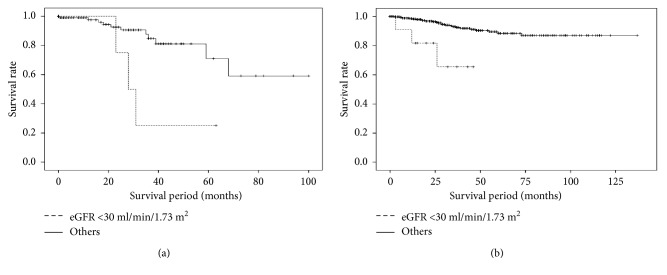
Comparison of the survival rate between patients whose estimated glomerular filtration rate (eGFR) was <30 ml/min/1.73 m^2^ and other patients in the elderly and nonelderly groups. (a) Elderly group. The mean survival time periods were 36.3 ± 7.9 and 78.2 ± 6.1 months among patients whose eGFR was <30 ml/min/1.73 m^2^ and among other patients, respectively (*P* < 0.05, log-rank test). (b) Nonelderly group. The mean survival time periods were 35.7 ± 5.0 and 124.1 ± 2.6 months among patients whose eGFR was <30 ml/min/1.73 m^2^ and among other patients, respectively (*P* < 0.05, log-rank test).

The results of univariate and multivariate analyses of the risk factors of death in all patients (elderly and nonelderly groups) are shown in Table 5. Overall, univariate analysis showed age, sex (male), eGFR <30 ml/min/1.73 m [2], CVD, and use of anticoagulants and/or antiplatelet drugs were significantly associated with the death after ESD. On the other hand, multivariate analysis showed that age and eGFR <30 ml/min/1.73 m [2] were independent risk factors of the death after ESD. In the nonelderly group, multivariate analysis showed BMI (<22 kg/m^2^) and eGFR <30 ml/min/1.73 m^2^ were risk factors of the death after ESD. In the elderly group, multivariate analysis showed that eGFR <30 ml/min/1.73 m [2] was an independent risk factor of death (hazard ratio = 5.32; 95% confidence interval = 1.39–20.5; *P*=0.015). There were 7 (3 died) and 13 (3 died) patients whose eGFR was <30 ml/min/1.73 m^2^ in the elderly and nonelderly groups, respectively. In the elderly group, of these 3 patients whose eGFR was <30 ml/min/1.73 m [2], 1 patient died from renal failure while cause of the death of the other 2 patients were unclear. In the nonelderly group, of these 3 patients whose eGFR was <30 ml/min/1.73 m [2], 1 patient died from multiple organ failure and 1 patient died from hepatocellular carcinoma, while cause of the death of the other 1 patient was unclear. The survival rates of the patients whose eGFR was <30 ml/min/1.73 m^2^ and other patients in both groups are shown in Figures 2(a) and 2(b), respectively. The mean survival time periods of the patients whose eGFR was <30 ml/min/1.73 m^2^ and other patients were 36.3 ± 7.9 and 78.2 ± 6.1 months (*P* < 0.05, log-rank test) in the elderly group and 35.7 ± 5.0 and 124.1 ± 2.6 months (*P* < 0.05, log-rank test) in the nonelderly group, respectively. There were 39 (2 died) and 141 (15 died) patients whose BMI was <22 kg/m^2^ in the elderly and nonelderly groups, respectively. The survival rates of the patients whose BMI was <22 kg/m^2^ and other patients in both groups are shown in Supplementary [Supplementary-material supplementary-material-1]. The mean survival time periods of the patients whose BMI was <22 kg/m^2^ and other patients were 93.3 ± 4.5 and 62.8 ± 5.8 months (not significant, log-rank test) in the elderly group and 91.8 ± 4.1 and 115.2 ± 2.2 months (*P* < 0.05, log-rank test) in the nonelderly group, respectively.

### 3.5. Characteristics of the Patients Who Died from Gastric Cancer and Their Lesions

The details of the patients who died from gastric cancer and their lesions are shown in Supplementary [Supplementary-material supplementary-material-1]. As previously described, in each group, 1 patient died from gastric cancer. One patient was a 74-year-old man with non-CR. He refused to undergo additional treatment and died 35 months after ESD. The other patient was an 80-year-old man with non-CR. He had myelodysplastic syndrome (MDS). Gastrectomy and lymphadenectomy were performed when local recurrence was detected 24 months after ESD. The pathology showed lymph node metastasis. However, adjuvant chemotherapy could not be performed because of MDS. He died 35 months after ESD.

## 4. Discussion

Some previous studies have analyzed OS and the risk factors of death among elderly patients with EGC after ESD [18–20]. Sumiyoshi et al. reported that the 5-year survival rates of elderly patients with EGC who underwent ESD after CR and non-CR with and without additional surgical resection were 84.6%, 73.3%, and 58.8%, respectively [18]. Yoshifuku et al. reported that during the follow-up period after ESD for EGC, the frequency of death was significantly higher in patients with low- and high-risk comorbidities than in those with no comorbidities [19]. However, to the best of our knowledge, no reports have examined OS in elderly patients with EGC after ESD with a focus on not only eGFR but also CKD staging, and therefore, the present study is novel. The survival rate was significantly shorter among patients whose eGFR was <30 ml/min/1.73 m^2^ than among other patients in both groups. Furthermore, eGFR <30 ml/min/1.73 m^2^ was an independent risk factor of death in the elderly group. Imai et al. reported that eGFR declines in accordance with aging [24]. Patients with CKD have a high risk of hospitalization for pneumonia, sepsis, and bacteremia [25]. Especially, patients with severe CKD have more risk of cancer development [26]. Furthermore, CKD stages G4 and G5 (eGFR <30 ml/min/1.73 m^2^) are more frequently associated with terminal renal dysfunction and mortality, as well as the incidence of CVD events, when compared to the incidence of these events for other stages of CKD [27, 28]. Special care should be taken when following up elderly patients with eGFR <30 ml/min/1.73 m^2^ who have a high risk of death during observation.

With regard to OS, the mean survival time was 75.8 ± 5.9 months in the elderly group. Considering the average life expectancy of male and female individuals in Japan [14], patients in the elderly group were assumed to live at least as long as the average life expectancy of Japanese individuals. In each of the study groups, 1 patient died from gastric cancer. Consequently, DSS was appropriate in both groups. Tsukuma et al. reported that the cumulative 5-year risk of EGC without any treatment progressing to the advanced stage was 63.0% [29], indicating that EGC generally grows slowly. However, once early gastric cancer grows up to advanced gastric cancer, the 3-year survival rate is 31% for those surgically treated and 0% for those observed conservatively [30]. These reports suggest the importance of ESD for EGC to achieve a high DSS rate.

Sekiguchi et al. reported that the 3-year (54.3% vs. 95.9%) and 5-year OS (54.3% vs. 76.3%) rates were significantly worse in patients with a low prognostic nutritional index among elderly patients who underwent ESD for EGC [20]. In this study, multivariate analysis showed that low BMI was a risk factor of death among nonelderly patients included. Therefore, we should consider a patient's metabolic status, such as low BMI and low nutritional condition, when performing follow-up after ESD.

The results of this study showed that there were no significant differences with regard to en block resection, CR, and adverse events of ESD between elderly and nonelderly patients. Gastric ESD for EGC in elderly patients aged ≥80 years can achieve high rates of curability and a low risk of adverse events, which are similar to those in nonelderly patients. These results were almost the same as the results of previous reports [16–19]. We could confirm that gastric ESD is a minimally invasive and effective treatment even for elderly patients who are prone to having several comorbidities.

Venous invasion-positive tumors were significantly more common in the elderly group than in the nonelderly group. The presence of papillary or undifferentiated-type adenocarcinoma was reported to be significantly associated with venous involvement [31]. In the elderly group, 3 of 6 venous invasion-positive lesions contained a papillary or undifferentiated adenocarcinoma component. However, only 1 of 5 lesions in the nonelderly group had a papillary adenocarcinoma component, and no lesion had an undifferentiated adenocarcinoma component. This histological difference between the 2 groups might have contributed to the high rate of venous invasion in the elderly group. Of note, for 1 lesion in the elderly group, venous invasion was detected although the tumor was a differentiated-type lesion and the depth was within the intramucosa. When performing gastric ESD for elderly patients, we should consider these factors and pay attention to the explanation provided to patients before ESD, as well as to the postoperative follow-up.

Additional surgical resection was performed in 2 (1.9%) patients in the elderly group and 24 (5.6%) patients in the nonelderly group. Considering the fact that each group had 1 patient who died from gastric cancer, additional surgical resection should not be discouraged even for elderly patients if they consent to the treatment and their general status is appropriate. Sekiguchi et al. developed and validated a risk-scoring model of lymph node metastasis in EGC [32]. This scoring model may be one of the helpful items to judge the adaptation of additional surgical resection.

The present study has some limitations. First, the study design was retrospective, and the study was performed in a single center. Second, the numbers of patients and lesions were relatively small. Third, the follow-up period was relatively short. The mean observation periods in this study were 26.9 and 36.4 months in elderly and nonelderly patients, respectively. These results were rather short compared to those of previously reported (39–41 months) [19, 21]. The risk of cancer recurrence might be estimated lower due to short observation period. In the future, prospective studies with a long-term follow-up are needed to evaluate the long-term outcomes of ESD for EGC in elderly patients.

In conclusion, ESD for EGC can be performed safely and can achieve high curability with a good prognosis in elderly patients aged ≥80 years. After ESD, close attention should be paid when following up elderly patients with severe CKD.

## Figures and Tables

**Table 1:** Clinical characteristics of patients in the elderly and nonelderly groups.

	Elderly group (*n* = 108)	Nonelderly group (*n* = 425)	*P* value
Age (mean ± SD), years	83.4 ± 2.7	69.6 ± 7.9	<0.05^*∗*^
Sex (male/female)	82/26	306/119	n.s.^*∗∗*^
BMI (mean ± SD), kg/m^2^	22.4 ± 3.0	23.2 ± 3.8	n.s.^*∗*^
ECOG-PS (0–1/2–4)	108/0	425/0	n.s.^*∗∗∗*^
Preoperative eGFR (mean ± SD), ml/min/1.73 m^2^	57.6 ± 16.8	69.4 ± 18.9	<0.05^*∗*^
Use of anticoagulants and/or antiplatelet drugs, *n* (%)	30 (27.8)	68 (16.0)	<0.05^*∗∗*^
Comorbidities, *n* (%)			
Cardiovascular disease	23 (21.3)	58 (13.6)	n.s.^*∗∗*^
History of cerebrovascular events	9 (8.3)	16 (3.8)	n.s.^*∗∗∗*^
Hypertension	54 (50.0)	162 (38.1)	<0.05^*∗∗*^
Diabetes	12 (11.1)	46 (10.8)	n.s.^*∗∗*^

SD, standard deviation; BMI, body mass index; PS, performance status; eGFR, estimated glomerular filtration rate. ^*∗*^Unpaired *t*-test; ^*∗∗*^chi-square test;^*∗∗∗*^Fisher's exact test.

**Table 2:** Clinical characteristics of the lesions in the elderly and nonelderly groups.

	Elderly group (*n* = 128)	Nonelderly group (*n* = 504)	*P* value
Macroscopic type, *n* (%)			
Elevated	58 (45.3)	223 (44.2)	n.s.^*∗*^
Flat/depressed	41 (32.0)	202 (40.1)	n.s.^*∗*^
Mixed	29 (22.7)	79 (15.7)	n.s.^*∗*^
Location, *n* (%)			
Upper	16 (12.5)	87 (17.3)	n.s.^*∗*^
Middle	35 (27.3)	143 (28.4)	n.s.^*∗*^
Lower	77 (60.2)	274 (54.3)	n.s.^*∗*^
Histological type, *n* (%)			
Differentiated	125 (97.7)	489 (97.0)	n.s.^*∗*^
Undifferentiated	3 (2.3)	15 (3.0)	n.s.^*∗∗*^
Tumor depth, *n* (%)			
Intramucosa	114 (89.0)	456 (90.5)	n.s.^*∗*^
SM1	7 (5.5)	26 (5.2)	n.s.^*∗∗*^
SM2	7 (5.5)	22 (4.3)	n.s.^*∗∗*^
Tumor size (mean ± SD), mm	16.5 ± 10.4	16.5 ± 11.8	n.s.^*∗∗∗*^
Venous invasion, *n* (%)	6 (4.7)	5 (1.0)	<0.05^*∗∗*^
Lymphatic vessel invasion, *n* (%)	5 (3.9)	17 (3.4)	n.s.^*∗∗*^

SD, standard deviation; SM1: depth of submucosal invasion <500 *μ*m; SM2: depth of submucosal invasion ≥500 *μ*m. ^*∗*^Chi-square *t*-test; ^*∗∗*^Fisher's exact test; ^*∗∗∗*^Unpaired *t*-test.

**Table 3:** Curative resection rates and complications related to endoscopic submucosal dissection.

	Elderly group (108 patients) (128 lesions)	Nonelderly group (425 patients) (504 lesions)	*P* value
En block resection, *n* (%)	125 (97.7)	482 (95.6)	n.s.^*∗*^
Curative resection, *n* (%)	110 (85.9)	432 (85.7)	n.s.^*∗∗*^
Noncurative resection (eCuraC1/eCuraC2), *n* (%)	18 (3/15)	72 (14/58)	n.s.^*∗∗*^
Patients who underwent additional surgical resection, *n* (%)	2 (1.9)	24 (5.6)	n.s.^*∗*^
Complications, *n* (%)			
Postoperative bleeding	7 (6.5)	18 (4.2)	n.s.^*∗*^
Perforation	0 (0)	1 (0.2)	n.s.^*∗*^
Aspiration pneumonia	4 (3.7)	11 (2.6)	n.s.^*∗*^
Delirium	1 (0.9)	0 (0)	n.s.^*∗*^

ESD, endoscopic submucosal dissection; eCuraC1, noncurative resection because horizontal margin was positive according to the Japanese gastric cancer treatment guidelines; eCuraC2, noncurative resection due to the factor other than eCuraC1 according to the Japanese gastric cancer treatment guidelines. ^*∗*^Fisher's exact test; ^*∗∗*^chi-square test.

**Table 4:** Long-term outcomes of endoscopic submucosal dissection.

	Elderly group (108 patients)	Nonelderly group (425 patients)	*P* value
Mean observation period (mean ± SD), months	26.9 ± 23.2	36.4 ± 29.6	<0.05^*∗*^
Gastric cancer death after ESD, *n* (%)	1 (0.9)	1 (0.2)	n.s.^*∗∗*^
Deaths caused by conditions other than gastric cancer, *n* (%)	13 (12.0)	28 (6.6)	0.06^*∗∗∗*^
Mean period from ESD to death, (mean ± SD), months	30.2 ± 16.6	26.8 ± 17.8	n.s.^*∗*^
Cause of death, *n* (%)^†^			
Renal dysfunction	2 (15.4)	0 (0)	<0.05^*∗∗*^
Cardiovascular disease	1 (7.7)	2 (7.1)	n.s.^*∗∗*^
Cerebrovascular events	2 (15.4)	2 (7.1)	n.s.^*∗∗*^
Pneumoniae	1 (7.7)	3 (10.7)	n.s.^*∗∗*^
Cancer (except for gastric cancer)	4 (30.8)	9 (32.2)	n.s.^*∗∗*^
Others	3 (23.0)	12 (42.9)	n.s.^*∗∗*^

ESD, endoscopic submucosal dissection; SD, standard deviation. ^†^The percentage was calculated according to the number of patients for each cause/deaths caused by conditions other than gastric cancer. ^*∗*^Unpaired *t*-test; ^*∗∗*^Fisher's exact test; ^*∗∗∗*^chi-square test.

**Table 5:** Univariate and multivariate analyses of the risk factors of death.

	Univariate analysis		Multivariate analysis	
	*P* value	HR (95% CI)	*P* value	HR (95% CI)
All patients				
Age	0.004	1.06 (1.02–1.10)	0.006	1.07 (1.02–1.12)
Sex (male)	0.074	2.09 (0.93–4.70)	0.10	2.25 (0.86–5.91)
BMI (<22 kg/m^2^)	0.085	1.83 (0.92–3.61)	0.076	1.91 (0.93–3.89)
Preoperative eGFR (<30 ml/min/1.73 m^2^)	<0.001	6.76 (2.82–16.20)	<0.001	8.09 (2.87–22.80)
Cardiovascular disease	0.016	0.80 (1.16–4.40)	0.18	2.10 (0.72–6.14)
History of cerebrovascular events	0.58	1.39 (0.43–4.50)	—	—
HT	0.48	0.79 (0.42–1.50)	—	—
Anticoagulants and/or antiplatelet drugs	0.029	2.03 (1.08–3.86)	0.586	0.74 (0.26–2.16)
Elderly group				
Age	0.73	1.02 (0.88–1.20)	—	—
Sex (male)	0.091	5.80 (0.76–44.59)	0.27	2.41 (0.50–11.54)
BMI (<22 kg/m^2^)	0.167	0.33 (0.07–1.59)	—	—
Preoperative eGFR (<30 ml/min/1.73 m^2^)	0.018	4.85 (1.32–17.85)	0.015	5.32 (1.39–20.5)
Cardiovascular disease	0.087	2.54 (0.88–7.37)	0.71	1.33 (0.31–5.72)
History of cerebrovascular events	0.25	2.48 (0.53–11.51)	—	—
HT	0.10	0.34 (0.09–1.24)		
Anticoagulants and/or antiplatelet drugs	0.05	2.94 (1.03–8.46)	0.34	2.03 (0.47–8.72)
Nonelderly group				
Age	0.13	1.04 (0.99–1.10)	—	—
Sex (male)	0.19	1.91 (0.73–5.00)	—	—
BMI (<22 kg/m^2^)	0.009	3.03 (1.33–6.93)	0.022	2.68 (1.15–6.24)
Preoperative eGFR (<30 ml/min/1.73 m^2^)	0.003	6.19 (1.84–20.88)	0.010	5.14 (1.49–17.70)
Cardiovascular disease	0.24	1.71 (0.70–4.20)	—	—
History of cerebrovascular events	0.76	0.74 (0.10–5.41)	—	—
HT	0.86	1.07 (0.50–2.27)		
Anticoagulants and/or antiplatelet drugs	0.38	1.47 (0.63–3.44)		

BMI, body mass index; eGFR, estimated glomerular filtration rate; CI, confidence interval; HT, hypertension.

## Data Availability

All data arising from this study are contained within the manuscript.

## Abbreviations

EGC: Early gastric cancer
ESD: Gastric endoscopic submucosal dissection
PS: Performance status
BMI: Body mass index
eGFR: Estimated glomerular filtration rate
CVD: Cardiovascular disease
CR: Curative resection
SM: Submucosa
OS: Overall survival
CKD: Chronic kidney disease
DSS: Disease-specific survival
MDS: Myelodysplastic syndrome.

## Supplementary Materials

Supplementary Materials Supplementary table 1: characteristics of the patients who died from gastric cancer and their lesions. Supplementary figure 1: cComparison of the survival rate between patients whose body mass index (BMI) was <22 kg/m^2^ and other patients in the elderly and nonelderly groups. Supplementary figure 1A: elderly group. The mean survival time periods were 93.3 ± 4.5 and 62.8 ± 5.8 months among patients whose BMI was <22 kg/m^2^ and among other patients, respectively (not significant, log-rank test). Supplementary figure 1B: nonelderly group. The mean survival time periods were 91.8 ± 4.1 and 115.2 ± 2.2 months among patients whose BMI was <22 kg/m^2^ and among other patients, respectively (*P* < 0.05, log-rank test).
