# Supplementary material for: Renal Dysfunction is a Risk Factor of Death after Gastric Endoscopic Submucosal Dissection in Elderly Patients Aged ≥80 Years
Source: Can J Gastroenterol Hepatol. 2019 Sep 9;2019:7145182. doi: 10.1155/2019/7145182 (PMC6754868; doi:10.1155/2019/7145182)
Supplement: Supplementary Materials — Supplementary table 1: characteristics of the patients who died from gastric cancer and their lesions. Supplementary figure 1: cComparison of the survival rate between patients whose body mass index (BMI) was <22 kg/m2 and other patients in the elderly and nonelderly groups. Supplementary figure 1A: elderly group. The mean survival time periods were 93.3 ± 4.5 and 62.8 ± 5.8 months among patients whose BMI was <22 kg/m2 and among other patients, respectively (not significant, log-rank test). Supplementary figure 1B: nonelderly group. The mean survival time periods were 91.8 ± 4.1 and 115.2 ± 2.2 months among patients whose BMI was <22 kg/m2 and among other patients, respectively (P < 0.05, log-rank test). [file 7145182.f1.docx]

**Supplementary table 1. Characteristics of the patients who died from gastric cancer and their lesions**

| **Sex** | **Age (years)** | **Macroscopic type** | **Histological type** | **Tumor size (mm)** | **Curability** | **Additional treatment** | **Prognosis** |
| --- | --- | --- | --- | --- | --- | --- | --- |
| M | 74 | Elevated | Differentiated | 12 | Non-CR  (SM2^†^, ly(+), v(+)) | No additional treatment | Died 35 months after ESD |
| M | 80 | Elevated | Differentiated | 30 | Non-CR  (HM(+), VM(+)) | Gastrectomy  Lymphadenectomy | Died 35 months after ESD |

ESD, endoscopic submucosal dissection; CR, curative resection; ly, lymphatic vessel invasion; v, venous invasion; HM, horizontal margin; VM, vertical margin^. †^SM2: depth of submucosal invasion ≥500 μm


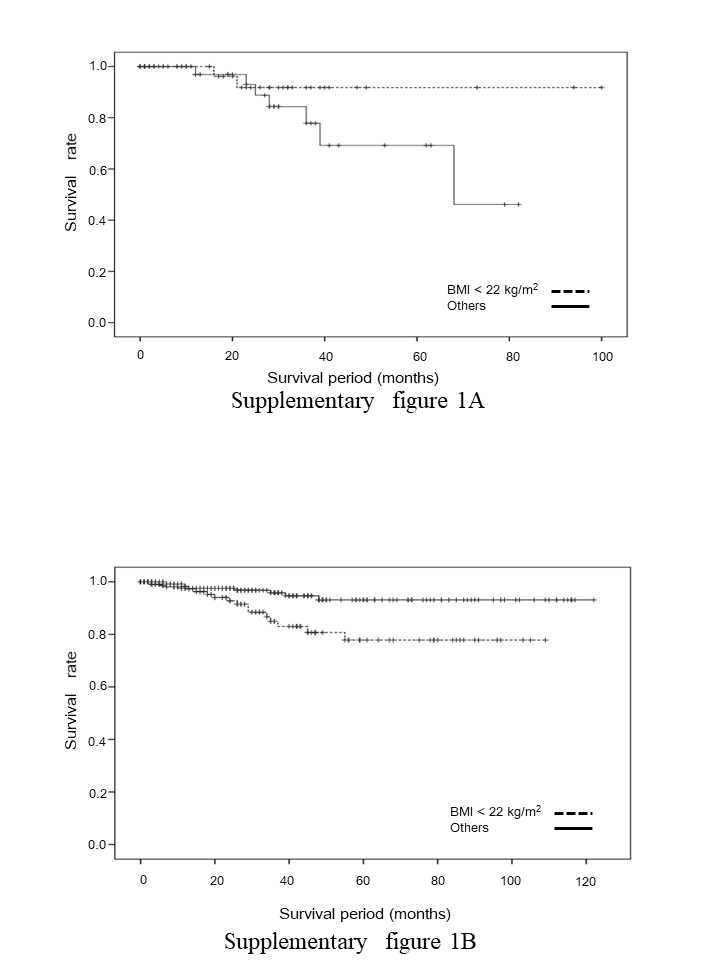


**Supplementary figure 1.** Comparison of the survival rate between patients whose body mass index (BMI) was <22 kg/m^2^ and other patients in the elderly and non-elderly groups.

**Supplementary figure 1A.** Elderly group. The mean survival times were 93.3 ± 4.5 and 62.8 ± 5.8 months among patients whose BMI was <22 kg/m^2^ and among other patients, respectively (not significant, log-rank test).

**Supplementary figure 1B.** Non-elderly group. The mean survival times were 91.8 ± 4.1 and 115.2 ± 2.2 months among patients whose BMI was <22 kg/m^2^ and among other patients, respectively (*P* < 0.05, log-rank test).
